# Methylisoindigo and Its Bromo-Derivatives Are Selective Tyrosine Kinase Inhibitors, Repressing Cellular Stat3 Activity, and Target CD133+ Cancer Stem Cells in PDAC

**DOI:** 10.3390/molecules22091546

**Published:** 2017-09-13

**Authors:** Jana Tegethoff, Roland Bischoff, Sawsan Saleh, Biljana Blagojevic, Karl-Heinz Merz, Xinlai Cheng

**Affiliations:** 1 Department of Pharmacy and Molecular Biotechnology, Division of Pharmaceutical Biology, University of Heidelberg, Im Neuenheimer Feld 364, D-69120 Heidelberg, Germany; jana.tegethoff@gmail.com (J.T.); sawsanalisaleh@gmail.com (S.S.); blagojevicbiljana@yahoo.com (B.B.); 2 Department of Chemistry, Division of Food Chemistry and Toxicology, University of Kaiserslautern, Erwin-Schrödinger-Strasse 52, D-67663 Kaiserslautern, Germany; roland@bischoffonline.de (R.B.); karlheinz_merz@yahoo.de (K.-H.M.)

**Keywords:** indirubin, meisoindigo, cancer stem cells, Stat3 inhibitor, selective protein kinase inhibitor, TCM, structure-activity-relationship

## Abstract

Indirubin is an active component of the herbal ingredient ‘Danggui Longhui wan’, which was used for the treatment of inflammation and chronic myeloid leukemia in China. The recent study showed its derivative methylisoindigo (also known as meisoindigo) preferentially targeting cancer stem cells (CSCs) in interference with AMPK and LKB1, the cellular metabolic sensors. In this study, we screened the effect of meisoindigo on a panel of 300 protein kinases and found that it selectively inhibited Stat3-associated tyrosine kinases and further confirmed its activity in cell based assays. To gain a deeper insight into the structure–activity relationship we produced 7 bromo-derivatives exhausting the accessible positions on the bisindole backbone except for in the 4-position due to the space limitation. We compared their anti-proliferative effects on tumor cells. We found that 6-bromomeisoindigo showed improved toxicity in company with increased Stat3 inhibition. Moreover, we detected that 6-bromomeisoindigo induced apoptosis of 95% of CD133+ pancreatic cancer cells. Considering that CD133 is a common marker highly expressed in a range of CSCs, our results imply the potential application of 6-bromomeisoindigo for the treatment of CSCs in different types of cancers.

## 1. Introduction

**Figure 1:**
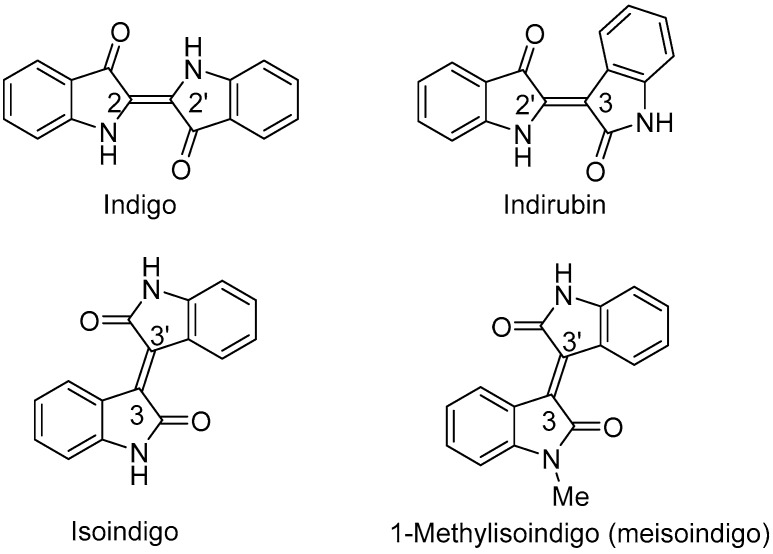
Structures of bisindoles: indigo, indirubin, isoindigo, and *N*-methylisoindigo (meisoindigo).

Indigo, a natural 2,2′-bisindole, is one of the most successful natural pigments with an annual consumption of several thousand tons (Figure 1) [1]. Its 3,2′-isoform, also known as indirubin, is used in China for treatment of chronic myeloid leukemia (CML). Clinical trials in the 1970s proofed the anti-leukemic effect of indirubin with mild side-effects [2]. However, the potential of indirubin for clinical application is limited by its extremely low water solubility. A number of follow-up studies were performed by either chemical modification [3,4,5] or lipid-based formulation [6,7,8] to improve its bioavailability. Isoindigo is a synthetic 3,3′-bisindole (Figure 1). Its *N*-methylated derivative (methylisoindigo, also known as meisoindigo) showed less side-effects and better bioavailability in animals and patients [2].

Results from recent studies indicated that the anti-tumorigenic effect of indirubin and its derivatives is majorly based on the potent inhibition of multiple protein kinases [9,10]. The identified target kinases include glycogen synthase kinase 3β (GSK-3β), cyclin-dependent kinases (CDKs) [9,11,12], CK2 [13], and IGF-1R [3]. By contrast, little is known about the effect of meisoindigo on protein kinases [14].

Recently, Hoheisel and his co-workers established a new primary pancreatic cell line, JoPaca-1, derived from a male patient suffering from pancreatic ductal adenocarcinoma [15]. This cell line contains a large population of cancer stem cells (CSCs) expressing stem cell markers CD133 [16,17]. Using this cell line as a model, we found that meisoindigo preferentially kills CD133 positive cells due to the inference with cellular metabolic signaling pathways involving AMPK and LKB1 [18]. CSCs are a sub-population of cancer cells that obtains the main features of stem cells: self-renewal, the capacity to strongly proliferate, and to develop into multiple lineages [18]. Former results revealed that they contribute to tumor initiation, formation of metastases, drug-resistances, and relapse in several cancers [19], like pancreatic cancer [20] and acute myeloid leukemia [21]. Thus, drugs inhibiting CSCs might be pioneering drugs for development of more efficient chemotherapy with expectations of eradicating metastases and preventing relapse [22].

In vitro and in vivo studies showed that indirubin derivatives are potent Src family kinases (SFKs) inhibitors [12,23,24,25,26,27,28]. Since SFKs are major kinases to regulate the activation of signal transducer and activator of transcription 3 (Stat3) [29], indirubin derivatives remarkably inhibit cellular Stat3 activity [26,27,28]. Stat3 is a key mediator of proliferation, cell cycle progression, angiogenesis, survival, and differentiation. Persistently active Stat3 has been found in various tumor types and tissues including leukemias [30], cervical [31], colorectal [32], and pancreatic cancer [33], which contributes to tumor initiation, progression, invasion, migration, and formation of metastases [34,35]. In addition, Stat3 plays an important role in the maintenance of stem cell-like properties such as self-renewal in embryonic stem cells and pancreatic CSCs [36,37].

Thus, Stat3 could be a potential target of meisoindigo involved in its negative regulation of CD133+ CSCs. In this work, we performed protein kinase profiling using a panel of 300 protein kinases and found that meisoindigo selectively inhibited tyrosine kinases related to Stat3 activity in vitro as well as in vivo, confirmed by cell-based assays. We investigated the structure–activity relationship by studying the anti-proliferative effects of newly synthesized novel bromo-meisoindigos. The results show that only 6-bromo-meisoindigo enhanced the inhibition on both Stat3 activity and cell proliferation, suggesting the limitation of access for chemical modification towards a protein kinase inhibitor.

## 2. Results and Discussion

### 2.1. Chemistry

**Scheme 1:**
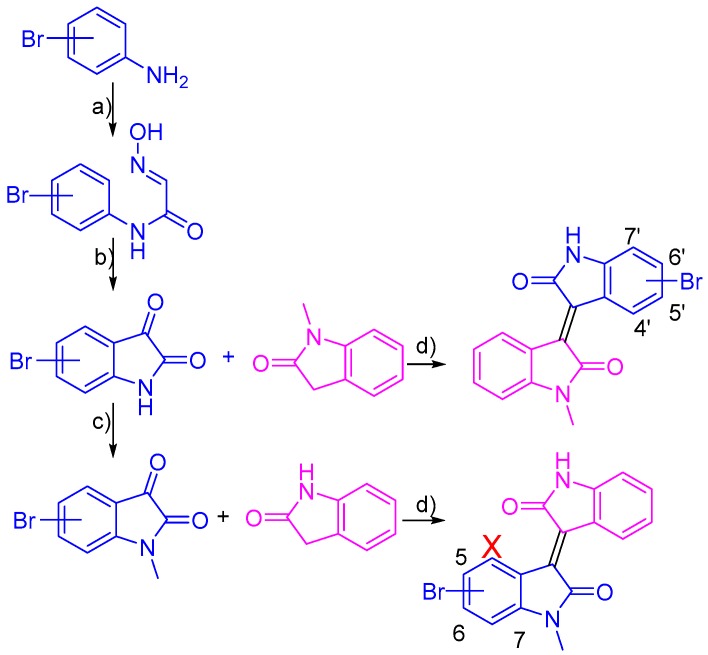
Synthesis of bromo-meisoindigo derivatives (**a**) CCl_3_CH(OH)_2_, NH_2_OH·HCl, H_2_O, Na_2_SO_4_; (**b**) H_2_SO_4_; (**c**) (CH_3_O)SO_2_, NaH, DMF; (**d**) HOAc/HCl 100:1, 120–128 °C, 3.5 h, X: unable to obtain the derivative in this position due to steric hindrance.

The lack of systematic evaluation of the chemical accessibility of positions on bisindole backbone inspired us to synthesize various bromo-meisoindigos. Those novel meisoindigo derivatives (**1**–**7**) were obtained by acidic condensation of corresponding bromoisatins with either methyloxindoles or methylated bromoisatins with oxindoles, as showed in Scheme 1 [13,18]. Bromo-isatins were synthesized by acidic cyclization of bromo-isonitrosoacetanilides achieved in the reaction of bromoanilines with chloral hydrate and hydroxylamine hydrochloride [3]. In the case of 3-bromoaniline, the formation of a mixture of 4- and 6-bromoisatins was observed and could be separated by fractionated precipitation. Bromoisatins reacted further with dimethylsulfate in dried dimethylformamide (DMF) containing NaH as base under nitrogen atmosphere to afford methylated products. Because of the steric hindrance, we produced 4′-bromo-meisoindigo in an extremely low yield (<10%) and failed to synthesize 4-bromo-1-meisoindigo. All chemical structures of compounds are listed in Table 1.

### 2.2. Biology

#### 2.2.1. Meisoindigo Is a Selective Stat3-Related Tyrosine Kinase Inhibitor

**Figure 2:**
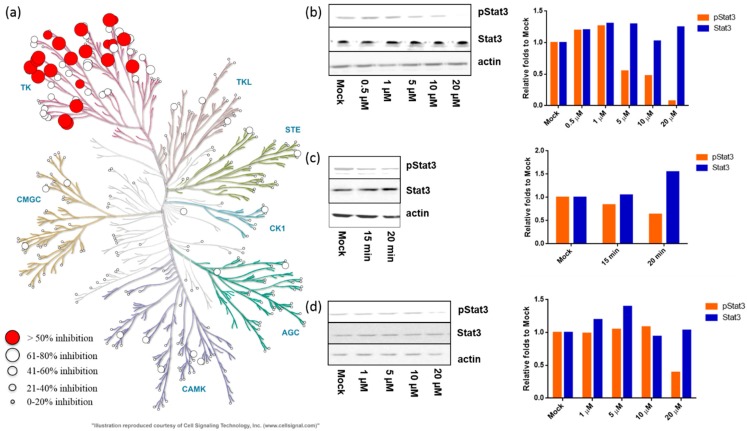
(**a**) Kinase profiling of meisoindigo at 20 µM against 300 human kinases. Circles show inhibited kinases, the size indicates the strength of inhibition. Kinases inhibited more than 50% are marked red including only tyrosine kinases (TKs). The graphic was generated using KinMap beta developed by BioMedX; (**b**) Meisoindigo inhibited Stat3 activation in HeLa cells in a dose-dependent manner. HeLa cells were incubated with meisoindigo for 30 min. The whole cell lysate was used for immunoblot. Specific antibodies against Stat3 and phospho-Stat3 (Y705) were applied. β-actin served as loading control and 0.1% DMSO as mock; (**c**) Meisoindigo inhibited Stat3 activation in HeLa cells in a time-dependent manner. Cells were treated at 5 µM as indicated and immunoblot was performed; (**d**) Meisoindigo inhibited Stat3 activation in JoPaca-1 cells in a dose-dependent manner. JoPaca-1 cells were treated for 2 h as indicated and immunoblot was performed.

Results from biological studies implied that meisoindigo might target distinct protein kinases in comparison to indirubin [9]. To gain a better insight in its mechanism of action we screened the inhibitory effect of meisoindigo in a panel of 300 human protein kinases. As shown in Figure 2a and supplementary information, meisoindigo (20 µM) was a highly selective tyrosine kinase inhibitor with a selective score of 0.07 (calculated as the number of kinases with residual activity <50% divided by 300). Interestingly, most of them are involved in the regulation of Stat3 activity [34,38,39], including growth factor-related receptor tyrosine kinases, insulin-like growth factor receptor 1 (IGF-R1), fibroblast growth factor receptor 2 (FGF-R2), vascular endothelia growth factor receptor 2 (VEGF-R2), and FGF-R3 [39,40], as well as Trk-B, Ron, Ret, and Mertk which activate Stat3 directly or via interleukin 6 and MAPK/Erk-associated signaling [41,42,43,44] and Src family kinases (SFKs), such as Lyn, Fyn, Src, Lck, Yes [29,45,46,47], Breast tumor kinase (BRK), Fes, and Fer [48,49,50].

#### 2.2.2. Meisoindigo Inhibits Cellular Stat3 Activation in Time and Concentration Dependent Manner

We investigated the influence of meisoindigo on Stat3 activity in HeLa cells by measuring the level of phospho-Stat3. The immunoblot result confirmed that meisoindigo inhibited cellular Stat3 up a concentration of 5 µM (Figure 2b), which occurred as early as 15 min upon treatment (Figure 2c). JoPaca-1 cells are more resistant and the reduction of phospho-Stat3 was observed upon treatment of 20 µM for 2 h (Figure 2d). In both cell lines pan-Stat3 remained unaffected. Given that persistent Stat3 activation drives tumor initiation and progression [34] and mediates stemness in pancreatic CSCs, the anti-proliferative effect of meisoindigo and its inhibition on CSCs reported previously [18] might partially depend on Stat3.

#### 2.2.3. Anti-Proliferative Effect of Novel Bromo-Meisoindigos

**Figure 3:**
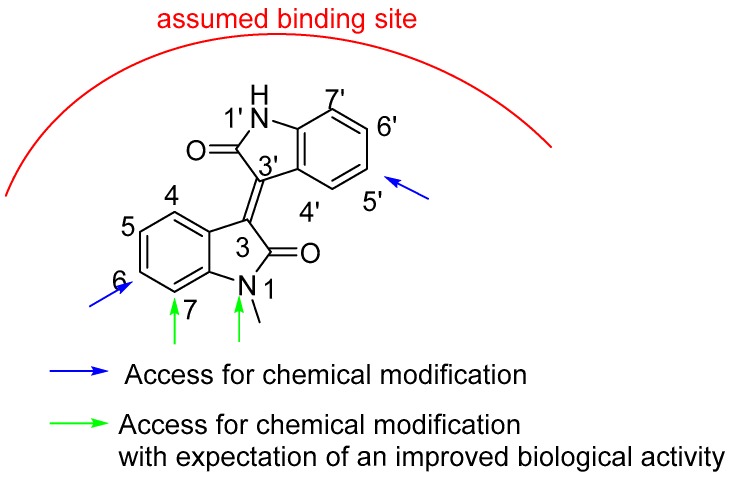
Assumed binding site and potential accesses for chemical modification of isoindigo.

We tested anti-proliferative effects of newly synthesized bromo-meisoindigos on HeLa, HCT116, and JoPaca-1 cells. Out of seven compounds 6-, 7- and 5′-bromo-meisoindigo showed the reduction of cellular viability with IC_50_ values lower than 50 µM in all three cell lines, while the introduction in the 5- and 7′-positions completely blocked the activity. It seems that the substituent in the 4-position interrupts the planar structure of meisoindigo and results in the inactivation. Comparison to the binding of indirubin to CDK2 in ATP-binding pocket [9], H-bridges might be formed between 1′-NH-CO of meisoindigos and target protein kinases (Figure 3), which could explain the strong impact caused by substitution in 5- and 7′-position. Moreover, we measured the toxicity of meisoindigo and its 6-bromo-derivative in human primary fibroblasts and found that both were not toxic at the concentration lower than 50 µM (Table 2), implying that meisoindigos preferentially target cancer cells. Thus, 6-Bromo-meisoindigo emerged as the most active compound and was selected for further analysis.

#### 2.2.4. 6-Bromo-Meisoindigo Inhibits Stat3 Activity and Induces Cell Cycle Arrest in HeLa Cells

**Figure 4:**
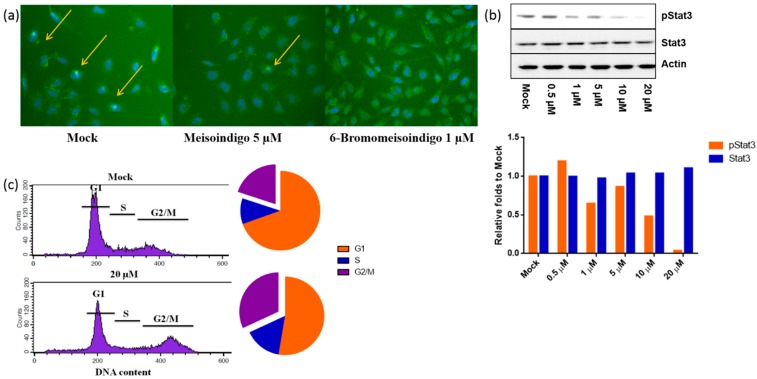
(**a**) Meisoindigo and 6-bromo-meisoindigo reduced Stat3 activity in HeLa cells. Cells were treated as indicated and fixed in formaldehyde. Specific antibodies against phospho-Stat3 (Y705) and Hoechst stain were used for immunocytochemistry assay; (**b**) 6-bromo-meisoindigo reduced Stat3 activity in HeLa cells in a dose-dependent manner. HeLa cells were incubated with compound for 30 min. The whole cell lysate was used for immunoblot. Specific antibodies against Stat3 and phospho-Stat3 (Y705) were applied. β-actin served as loading control and 0.1% DMSO as mock; (**c**) 6-bromo-meisoindigo induced a G2/M cell cycle arrest in HeLa cells, determined by FACS analysis.

Upon phosphorylation at tyrosine 705 (Y705), Stat3 is activated, dimerizes, and translocates into the nucleus to promote downstream gene expression [1,38,39]. Thus, nucleic translocation of cytosolic Stat3 is of importance for activation of Stat3 downstream gene expression [51]. We recruited immunocytochemistry and clearly showed the reduction of nucleic phospho-Stat3 upon treatment with either meisoindigo (5 µM) or 6-bromo-meisoindigo (1 µM) in HeLa cells (Figure 4a). Immunoblot further confirmed the reduction in the level of total cellular phospho-Stat3 up a concentration of 1 µM (Figure 4b), while pan-Stat3 remained constant. Since Stat3 regulates cell cycle progression [52], we determined cell cycle progression in living HeLa cells using FACS (fluorescence-activated cell sorting) analysis. In the presence of 6-bromo-meisoindigo, cells accumulated in the G2 and M phases instead of progressing to G1 phase (Figure 4c).

#### 2.2.5. 6-Bromo-Meisoindigo Inhibits Stat3 Activity in Jopaca-1 Cells

**Figure 5:**
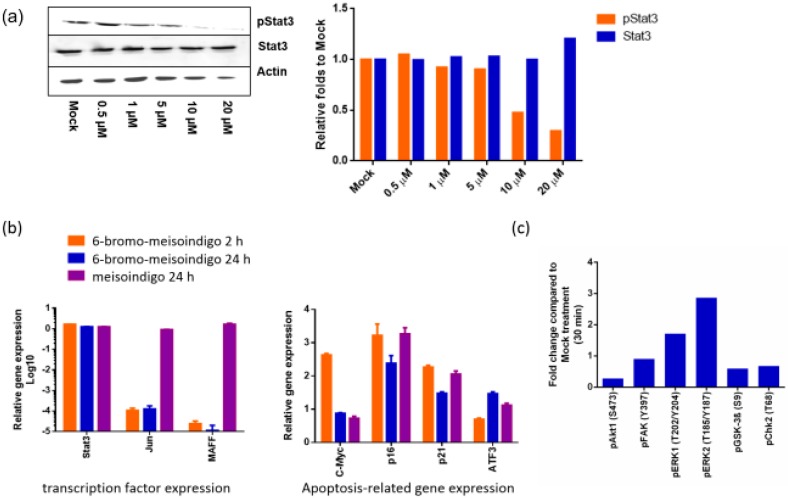
(**a**) 6-Bromo-meisoindigo inhibits Stat3 activation in JoPaca-1 cells in a dose-dependent manner. Cells were incubated with compound for 30 min. The whole cell lysate was used for immunoblot. Specific antibodies against Stat3 and phospho-Stat3 (Y705) were applied. β-actin served as loading control and 0.1% DMSO as mock; (**b**) 6-bromo-meisoindigo altered expression of Stat3 related transcription factors and apoptosis-related genes; (**c**) Protein microarray analysis of phosphorylation of protein kinases in 6-bromo-meisoindigo-treated Jopaca-1 cells.

We analyzed Stat3 activity in JoPaca-1 cells in the presence/absence of 6-bromo-meisoindigo by immunoblot and found 10 µM was sufficient to block phospho-Stat3 (Figure 5a). qRT-PCR using cDNA synthesized with random primers from RNA isolated from 6-bromo-meisoindigo-treated Jopaca-1 cells exhibited a clear reduction in the level of c-Jun and MAFF expression (Figure 5b), downstream genes of Stat3 [53], while Stat3 expression was unaffected. c-Jun is a transcription factor that mediates progression through G2/M phase and inhibition of apoptosis [54,55], in consistence that 6-bromo-meisoindigo induced cell-cycle arrest at G2/M in HeLa cells. MAFF is a transcription factor that regulates oxidative stress response [56], suggesting disruption of cellular homeostasis might be involved in meisoindigo and derivatives-induced apoptosis, like we showed previously [18].

We also compared the level of four apoptosis-related genes expression (Figure 5b). c-Myc, an oncogene that is overactive in numbers of tumors and contributes to tumor initiation and progression [57], was slightly suppressed in presence of 6-bromo-meisoindigo or meisoindigo for 24 h. p16 and p21 are CDK inhibitors and play important roles in the regulation of cell proliferation. Expression of both was strongly increased by 6-bromo-meisoindigo and meisoindigo suggesting that observed cell cycle arrest at least partially depended on their inhibition of CDKs. ATF3 is another apoptosis related gene and inhibits the activity of p53, a key mediator of apoptosis [58]. The expression of ATF3 was reduced upon treatment with 6-bromo-meisoindigo for two hours. Results from phosphor-protein kinase microarray analysis (Figure 5c) confirmed that the activity of GSK3ß and FAK was not affected by meisoindigo derivatives, while reduction of phosphor-Akt was observed probably due to the inhibition of IGF-1R (Figure 2a and supplementary information). Thus, ATF3 may play a role in the induction of apoptosis by meisoindigos. Taken together, we showed that 6-bromo-meisoindigo inhibited Stat3 activity in JoPaca-1 cells and altered the expression of Stat3 related genes involved in the regulation of apoptosis and cell cycle.

#### 2.2.6. 6-Bromo-Meisoindigo Induces Apoptosis in CD133+ Jopaca-1 Cells

**Figure 6:**
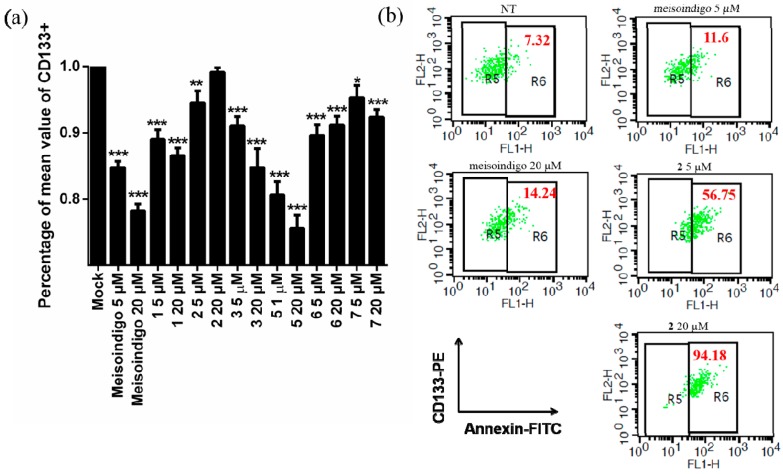
(**a**) Bromo-meisoindigos reduced CD133+ population in JoPaca-1 cells. The percentage of CD133 expression was determined by FACS analysis and the mean value was normalized to mock treatment; (**b**) Meisoindigo and 6-bromo-meisoindigo induced apoptosis in JoPaca-1 cells. Cells were treated as indicated for 24 h and FACS analysis was performed.

Our recent results showed that meisoindigo suppressed CD133 expression, preferentially killed CD133+ cells, and thereby impacted on the stemness of CSCs in JoPaca-1 cells [18]. We examined the effect of newly synthesized bromo-meisoindigos on CD133+ cells in JoPaca-1 and found the general inhibition of CD133 expression in the presence of bromo-meisoindigos (Figure 6a). Among those, 5′-bromo-meisoindigo (Figure 6a) showed the highest activity. Previous results demonstrated that CD133+ CSCs are very resistant to chemotherapeutic agents [15]. We interestingly found that nearly 60% and 90% of CD133+ CSCs were annexin v positive in the presence of 6-bromo-meisoindigo at 5 and 20 µM (Figure 6b), implicating the high potential of 6-bromo-meisoindigo for treatment of CD133+ CSCs, which might depend on its Stat3 inhibition.

## 3. Experimental Section

### 3.1. Materials

pStat3Tyr705 (9145), Stat3 (9139) primary antibodies, and Alexa Flour 488 Phalloidin (8878) were purchased from Cell Signaling Technology (Danvers, MA, USA). β-actin primary antibody and HRP-coupled secondary anti-mouse and anti-rabbit antibodies were from Santa Cruz Biotechnology (Santa Cruz, CA, USA). Fluorochrome-coupled Goat IgG anti-rabbit IgG Alexa Flour 594 was bought from dianova (Hamburg, Germany). DMEM, RPMI 1640 medium, fetal bovine serum (FBS), Penicillin/Streptomycin (Pen/Strep) and PBS were purchased from Gibco by Life Technologies (Carlsbad, CA, USA).

### 3.2. Synthesis of Bromo-Meisoindigo Derivatives

A mixture of 2-bromoaniline or 3-bromoaniline (110 mmol, 18.9 g), chloral hydrate (118.8 mmol, 19.6 g), anhydrous sodium sulfate (880 mmol, 125 g), and hydroxylamine hydrochloride (347.6 mmol, 24.2 g) in water (700 mL) was heated under reflux for 25 min to form a yellow precipitate. 50 mL of ethanol was added after that and left to boil for another 4 min before filtration. The resulted isonitrosoacetanilide was then heated with sulfuric acid (150 mL) at 70 °C for about 10 min then poured into ice water to form an orange precipitate. When using 3-bromoaniline, an extra step of separation is required by solving the resulted mixture in 0.25 M NaOH (1 L), filtering it, then adding acetic acid (100 mL) to afford 4-bromoistain as a precipitate. 6-Bromoistin can be then obtained when lowering the pH of the solution to 1 by hydrochloric acid 37%.

All substituted bromo-isatins were methylated by stirring (22 mmol, 4.97 g) with sodium hydride (2 mmol, 0.968 g) and dimethylsulfate (23.1 mmol, 2.19 mL) in dried DMF (50 mL) under nitrogen atmosphere. The resulting mixture was poured after 2 h in ice water (100 mL) to form an orange precipitate.

The methylated bromo-isatins (2 mmol, 0.480 g) were treated after that with oxindole (2 mmol, 0.226 g) in a 100:1 mixture of glacial acetic acid and hydrochloric acid 37% (12 mL) in reflux for about 3 h. Then, they were poured in ice water (50 mL) to afford 5-, 6- and 7-bromomeisoindigo. While 4′-, 5′-, 6′- and 7′-bromomeisoindigo were obtained by reacting unmethylated bromo-isatins (2 mmol, 0.452 g) with 1-methyloxindole (2 mmol, 0.294 g) in the same conditions. The resulted precipitates were washed with water, ethanol (2 × 10 mL), and diethylether (2 × 10 mL) then dried.

#### 3.2.1. 5-Bromo-1-Meisoindigo

Yield: 70%. ^1^H-NMR (400 MHz, DMSO, *d*_6_) δ ppm 3.19 (s, 3H), 6.83 (dd, ^3^*J* = 7.8 Hz, ^4^*J* = 0.5 Hz, 1H) 6.96 (m, 1H), 6.97 (d, ^3^*J* = 8.4 Hz, 1H), 7.35 (td, ^3^*J* = 7.6 Hz, ^4^*J* = 1.1 Hz), 7.58 (dd, ^3^*J* = 8.4 Hz, ^4^*J* = 2.1 Hz), 9.06 (dd, ^3^*J* = 8.1 Hz, ^4^*J* = 0.5 Hz), 9.31 (d, ^4^*J* = 2.0 Hz, 1H), 10.94 (s, 1H). ^13^C-NMR (100 MHz, DMSO, *d*_6_) δ ppm 26.6, 110.2, 110.7, 114.0, 121.8, 121.9, 123.0, 130.2, 131.1, 131.5, 133.8, 134.8, 135.6, 144.4, 145.0, 167.2, 169.3. Anal. calcd. for C_17_H_11_BrN_2_O_2_: C 57.49, H 3.12, N 7.89; found: C 57.44, H 3.18, N 793. HRMS (ESI) calculated *m*/*z*: 376.9896; found C_17_H_11_BrN_2_O_2_; *m*/*z*: 376.9896 [M + H]^+^.

#### 3.2.2. 6-Bromo-1-Meisoindigo

Yield: 83%.^1^H-NMR (400 MHz, DMSO, *d*_6_) δ ppm 3.17 (s, 3H), 6.79 (dd, ^3^*J* = 7.7 Hz, ^4^*J* = 0.5 Hz, 1H), 6.93 (td, ^3^*J* = 7.8 Hz, ^4^*J* = 1.1 Hz 1H), 7.17 (dd, ^3^*J* = 8.5 Hz, ^4^*J* = 2.0 Hz, 1H), 7.21 (d, ^4^*J* = 1.9 Hz, 1H), 7.32 (td, ^3^*J* = 7.6 Hz, ^4^*J* = 1.2 Hz, 1H), 8.97 (d, ^3^*J* = 8.5 Hz, 1H), 9.02 (dd, ^3^*J* = 7.6 Hz, ^4^*J* = 0.6 Hz, 1H), 10.9 (s, 1H). ^13^C-NMR (100 MHz, DMSO, *d*_6_) δ ppm 26.3, 109.7, 111.5, 119.9, 121.3, 124.3, 125.6, 129.5, 130.3, 130.9, 133.1, 134.3, 144.4, 146.2, 167.1, 168.9. Anal. calcd. for C_17_H_11_BrN_2_O_2_: C 57.49, H 3.12, N 7.89; found: C 57.43, H 3.25, N 7.87. HRMS (ESI) calculated *m*/*z*: 376.9896; found C17H11BrN2O2; *m*/*z*: 376.9896 [M + H]^+^.

#### 3.2.3. 7-Bromo-1-Meisoindigo

Yield: 40%. ^1^H-NMR (400 MHz, DMSO, *d*_6_) δ ppm 3.53 (s, 3H), 6.8 (d, ^3^*J* = 7.7 Hz, 1H), 6.92 (t, ^3^*J* = 8.0 Hz, 1H), 6.93 (m, 1H), 7.23 (td, ^3^*J* = 7.7 Hz ^4^*J* = 0.9 Hz, 1H), 7.52 (dd, ^3^*J* = 8.0 Hz, ^4^*J* = 0.7 Hz, 1H), 8.92 (d, ^3^*J* = 8.0 Hz, 1H), 9.07 (dd, ^3^*J* = 7.9 Hz, ^4^*J* = 0.8 Hz, 1H), 10.9 (s, 1H). ^13^C-NMR (100 MHz, DMSO, *d*_6_) δ ppm 29.7, 101.3, 109.7, 121.2, 121.6, 123.0, 124.0, 127.9, 129.6, 130.6, 133.4, 135.1, 137.3, 141.6, 144.5, 167.5, 168.5. Anal. calcd. for C_17_H_11_BrN_2_O_2_: C 57.49, H 3.12, N 7.89; found: C 57.86, H 3.03, N 7.91. HRMS (ESI) calculated *m*/*z*: 376.9896; found C17H11BrN2O2; *m*/*z*: 376.9896 [M + H]^+^.

#### 3.2.4. 4′-Bromo-1-Meisoindigo

Yield: 3.5%. ^1^H-NMR (400 MHz, DMSO, *d*_6_) δ ppm 3.2 (s, 3H), 6.83 (dd, ^3^*J* = 7.4 Hz, ^4^*J* = 1.1 Hz, 1H), 7.03 (d, ^3^*J* = 7.8 Hz, 1H), 7.05 (td, ^3^*J* = 8.1 Hz ^4^*J* = 1.0 Hz, 1H), 7.16 (dd, ^3^*J* = 8.1 Hz, ^4^*J* = 1.0 Hz, 1H), 7.21 (m, 1H), 7.45 (td, ^3^*J* = 7.8 Hz, ^4^*J* = 1.0 Hz, 1H), 8.62 (d, ^3^*J* = 7.6 Hz, 1H), 10.93 (s, 1H). ^13^C-NMR (100 MHz, DMSO, *d*_6_) δ ppm 26.3, 108.7, 108.8, 121.2, 121.8, 122.4, 123.4, 126.3, 129.5, 131.7, 132.7, 133.0, 133.5, 145.4, 146.2, 165.1, 168.8. HRMS (ESI) calculated *m*/*z*: 376.9896; found C_17_H_11_BrN_2_O_2_; *m*/*z*: 376.9896 [M + H]^+^.

#### 3.2.5. 5′-Bromo-1-Meisoindigo

Yield: 80%. ^1^H-NMR (400 MHz, DMSO, *d*_6_) δ ppm 3.21 (s, 3H), 6.97 (d, ^3^*J* = 8,3 Hz, 1H), 7.01 (d, ^3^*J* = 7.0 Hz, 1H), 7.03 (m, 1H), 7.44 (td, ^3^*J* = 7.7 Hz, ^4^*J* = 1.2 Hz, 1H), 7.50 (dd, ^3^*J* = 8.3 Hz, ^4^*J* = 2.1 Hz, 1H), 9.08 (dd, ^3^*J* = 8.0 Hz, ^4^*J* = 0.6 Hz, 1H), 9.33 (d, ^4^*J* = 2.0 Hz, 1H), 11.04 (s, 1H). ^13^C-NMR (100 MHz, DMSO, *d*_6_) δ ppm 26.6, 109.1, 111.8, 113.3, 121.0, 122.3, 123.8, 129.8, 131.9, 132.7, 133.6, 134.1, 135.2, 143.6, 145.8, 167.7, 168. Anal. calcd. for C_17_H_11_BrN_2_O_2_: C 57.49, H 3.12, N 7.89; found: C 57.34, H 2.91, N 7.75, HRMS (ESI) calculated *m*/*z*: 376.9896; found C_17_H_11_BrN_2_O_2_; *m*/*z*: 376.9896 [M + H]^+^.

#### 3.2.6. 6′-Bromo-1-Meisoindigo

Yield: 86%. ^1^H-NMR (400 MHz, DMSO, *d*_6_) δ ppm 3.17 (s, 3H), 6.92 (d, ^4^*J* = 1.9 Hz, 1H), 6.95 (d, ^3^*J* = 7,8 Hz, 1H), 7.00 (m, 1H), 7.11 (dd, ^3^*J* = 8.6 Hz, ^4^*J* = 2.0 Hz, 1H), 7.40 (td, ^3^*J* = 7.7 Hz, ^4^*J* = 0.9 Hz, 1H), 8.97 (d, ^3^*J* = 8.6 Hz, 1H), 9.03 (d, ^3^*J* = 7.7 Hz, 1H), 11.0 (s, 1H). ^13^C-NMR (100 MHz, DMSO, *d*_6_) δ ppm 26.0, 108.5, 112.2, 120.7, 121.8, 123.8, 125.5, 129.1, 130.9, 132.3, 132.8, 132.9, 145.1, 145.4, 167.1, 168.6. Anal. calcd. for C_17_H_11_BrN_2_O_2_: C 57.49, H 3.12, N 7.89; found: C 57.65, H 3.20, N 7.91. HRMS (ESI) calculated *m*/*z*: 376.9896; found C_17_H_11_BrN_2_O_2_; *m*/*z*: 376.9896 [M + H]^+^.

#### 3.2.7. 7′-Bromo-1-Meisoindigo

Yield: 73%. ^1^H-NMR (400 MHz, DMSO, *d*_6_) δ ppm 3.21 (s, 3H), 6.94 (d, ^3^*J* = 8.1 Hz, 1H), 7.03 (d, ^3^*J* = 7.6 Hz, 1H), 7.05 (m, 1H, 7.45 (td, ^3^*J* = 7.6 Hz, ^4^*J* = 0.8 Hz, 1H), 7.55 (dd, ^3^*J* = 7.9 Hz, ^4^*J* = 0.5 Hz, 1H), 9.06 (d, ^3^*J* = 7.8 Hz, 1H), 9.10 (dd, ^3^*J* = 8.0 Hz, ^4^*J* = 0.6 Hz, 1H), 11.16 (s, 1H). ^13^C-NMR (100 MHz, DMSO, *d*_6_) δ ppm 26.1, 101.9, 108.6, 120.7, 121.9, 122.6, 123.3, 128.2, 129.2, 133.0, 133.2, 133.8, 135.1, 143.0, 145.3, 167.0, 168.6. Anal. calcd. for C_17_H_11_BrN_2_O_2_: C 57.49, H 3.12, N 7.89; found: C 57.68, H 3.14, N 7.90. HRMS (ESI) calculated *m*/*z*: 376.9896; found C17H11BrN2O2; *m*/*z*: 376.9896 [M + H]^+^.

### 3.3. Cell Culture

HeLa and HCT116 were cultured in DMEM containing 10% fetal bovine serum (FBS) and 1% Penicillin/Streptomycin (Pen/Strep). JoPaca-1 cells were cultivated in RPMI 1640 medium supplemented with 10% FBS and 1% Pen/Strep. Cells were kept under 5% CO_2_ at 37 °C in a humidified atmosphere. Cells were treated with drugs solved in DMSO from Sigma-Aldrich (Germany) 24 h after seeding.

Human primary fibroblasts were isolated and cultivated as described previously with positive ethic permission [59,60].

### 3.4. Protein Kinase Profiling

Protein kinase profiling was performed by ProQinase (Freiburg, Germany) as previously reported [5]. The kinase map was generated using KinMap beta developed by BioMedX (Heidelberg, Germany).

### 3.5. Western Blotting

As previously reported [18,59,60], 2 × 10^5^ cells were seeded and treated with compounds for 30 min or 2 h. Cells were lysed in Urea-lysis buffer containing 1 mM EDTA, 0.5% Triton X-100, 5 mM NaF, 6 M Urea, 1 mM Na_3_VO_4_, 10 mg/mL Pepstatin, 100 mM PMSF, and 3 mg/mL Aprotinin in PBS. Protein concentrations were normalized to the smallest value and proteins were resolved on 8% SDS-PAGE and blotted to membrane using BlueFlash Large semi-dry blotter from Serva Electrophoresis (Heidelberg, Germany). The membrane was blocked for at least 2 h in 5% non-fat milk purchased form Roth (Karlsruhe, Germany) in TBS-T. For immunoblotting, all antibodies were applied according to manufacturers’ recommendations. Immunoblots were developed with ECL solution obtained from PerkinElmar (Waltham, MA, USA) and imaged using digital imaging system LAS-3000 from Fujifilm (Tokyo, Japan).

### 3.6. Cytotoxicity Assays

5 × 10^3^ cells/well were seeded and treated in quadruplicates with compounds at concentrations ranging from 0.4 µM to 100 µM for 24, 48 or 72 h. Cells were incubated for 2 h in medium containing 1% FBS and 0.5 mg/mL 3-(4,5-dimethylthiazol-2-yl)-2,5-diphenyltetrazolium bromide (MTT) bought from Sigma-Aldrich, washed in PBS, and incubated in DMSO from Honeywell (Morris Plains, NJ, USA) for 10 min shaking. Absorption was measured photometrically at 595 nm using Infinite F200 pro microplate reader from Tecan (Männedorf, Switzerland).

### 3.7. CD133 and Annexin V Staining

2 × 10^5^ cells/well were seeded and treated for 24 h with compounds. Cell were trypsinized and resuspended in 40 µL blocking buffer obtained from Beckton, Dickinson and Company (Franklin Lakes, NJ, USA) and incubated for 10 min in the dark. 10 μL staining solution containing CD133 antibody purchased from Miltenyi Biotec (Bergisch Gladbach, Germany) and Annexin V-FITC from eBioscience (San Diego, CA, USA) 1:10 in blocking buffer were added to each sample, which were then incubated for 15 min in the dark at room temperature. Finally, 450 mL FACS buffer obtained from Beckton, Dickinson and Company were added and the samples were immediately analyzed with a FACSCalibur device from Beckton, Dickinson and Company.

### 3.8. Immunocytochemistry

5 × 10^3^ cells/well were seeded and treated with compounds for 24 h. Cells were fixed in 4% formaldehyde (formaldehyde 37% from Merck (Darmstadt, Germany) in PBS) for 12 min and blocked for 60 min in blocking buffer (5% goat serum, 1% BSA, 0.3% Triton X-100 in PBS). For immunostaining, all antibodies were applied according to manufacturers’ recommendations. Photos were taken with Keyence BZ 9000 fluorescence microscope.

### 3.9. qRT-PCR

2 × 10^5^ cells/well were seeded and treated with compounds for 2 or 24 h. RNA was isolated using NucleoSpin RNA Kit obtained from Macherey-Nagel (Düren, Germany) and cDNA synthesis was performed with ProtoScript II First Strand cDNA synthesis Kit using standard protocol from New England Biolabs (Ipswich, MA, United States). qPCR was performed according to manufacturers’ recommendations in LightCycler 96 from Roche (Basel, Switzerland). Actin was used as reference gene for relative expression analysis which was performed as reported previously [61]. All primers were obtained from Eurofins Genomics (Luxembourg).The following primer pairs were used: ATF3 (5s: TCGGAGAAGCTGGAAAGTGT, 3as: TCTGGAGTCCTCCCATTCTG), c-Myc (5s: CCTGGCAAAAGGTCAGAGTC, 3as: GCTGCGTAGTTGTGCTGATG), IL-4 (5s: TTTGCTGCCTCCAAGAACAC, 3as: GTCGAGCCGTTTCAGGAATC), IL-6 (5s: AGACAGCCACTCACCTCTTC, 3as: AGTGCCTCTTTGCTGCTTTC), c-Jun (5s: TGAAACAGAGCATGACCCTG, 3as: GATTATCAGGCGCTCCAGCTC), MAFF (5s: TCTGTGGATCCCCTATCCAG, 3as: TCTGTGGATCCCCTATCCAG), p16 (5s: GAGCAGCATGGAGCCTTC, 3as: CATCATCATGACCTGGATCG), p21 (5s: GACACCACTGGAGGGTGACT, 3as: CAGGTCCACATGGTCTTCCT), β-actin (5s: CTGACTACCTCATGAAGATCCTC, 3as; CATTGCCAATGGTGATGACCTG), Stat3 (5s: AGATGCAGCAGCTGGAACAGAT, 3as: CGTGAGAGTTTTCTGCACGT).

### 3.10. Protein Microarray Analysis

200,000 JoPaca-1 cells were seeded and treated for 30 min with 6-bromo-meisoindigo at the concentration of 10 µM and 0.1% DMSO for mock treatment. Samples were collected and measured following ELISA-based microarray protocol previously described in Holenya et al. and using microarrays based on the ArrayStrip™ platform (Alere Technologies GmbH, Jena, Germany) [62,63]. The quantity of phosphorylated proteins (ng/µg of total protein) in 2-treated JoPaca-1 cells was compared to mock treatment and showed as fold change.

## 4. Conclusions

In this work, we identified that meisoindigo and 6-bromo-meisoindigo are potent Stat3 inhibitors and kill CD133+ CSCs in tumors. Given that Stat3 and CD133 are of importance in the regulation of proliferation and development of chemotherapeutic resistance [22,34], our results suggest meisoindigos as promising anti-cancer agents. The recent results demonstrated that active Stat3 led to the expression of immunosuppressive protein PD-L1, the major ligand of programmed death 1 (PD-1) [64]. It is very interesting if the combination of meisoindigos with a PD-1 or PD-L1 inhibitor can elicit a synergistic effect for immunotherapy. Moreover, our structure–activity relationship study also pinpoints that the 1- and 7-positions are available positions for further chemical modification.

## Figures and Tables

**Table 1:**
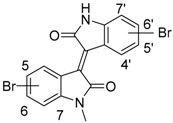
Chemical structures of novel-bromomeisoindigos.

Nr	Compounds	Nr.	Compounds
		**4**	4′-bromo-meisoindigo
**1**	5-bromo-meisoindigo	**5**	5′-bromo-meisoindigo
**2**	6-bromo-meisoindigo	**6**	6′-bromo-meisoindigo
**3**	7-bromo-meisoindigo	**7**	7′-bromo-meisoindigo

**Table 2:** IC50 values of compounds in HeLa, HCT116, and JoPaca-1 cells tested by MTT assay.

IC_50_/Cell Line		Meisoindigo	5-Bromo-Meisoindigo	6-Bromo-Meisoindigo	7-Bromo-Meisoindigo	4′-Bromo-Meisoindigo	5′-Bromo-Meisoindigo	6′-Bromo-Meisoindigo	7′-Bromo-Meisoindigo
HeLa	72 h	9.53 ± 1.09	>100	5.84 ± 0.40	24.69 ± 6.64	>50	18.32 ± 3.22	17.71 ± 1.98	>100
HCT116	72 h	9.09 ± 1.13	>100	7.55 ± 1.05	26.34 ± 9.63	n.t.	21.96 ± 3.22	16.17 ± 1.73	>100
JoPaca-1	72 h	13.92 ± 1.39	>100	10.52 ± 1.67	13.15 ± 0.99	>50	13.04 ± 1.49	>100	>100
HF	72 h	>50	n.t.	>50	n.t.	n.t.	n.t.	n.t.	n.t.

n.t.: not tested.

## Supplementary Materials

The supplementary information is available online.
