# Supplementary material for: Methylisoindigo and Its Bromo-Derivatives Are Selective Tyrosine Kinase Inhibitors, Repressing Cellular Stat3 Activity, and Target CD133+ Cancer Stem Cells in PDAC
Source: Molecules. 2017 Sep 13;22(9):1546. doi: 10.3390/molecules22091546 (PMC6151689; doi:10.3390/molecules22091546)
Supplement: Supplementary file 1 [file molecules-22-01546-s001.pdf]

Molecules **2014**, *19*, 1-x manuscripts; doi:10.3390/molecules190x0000x

OPEN ACCESS

molecules

ISSN 1420-3049

www.mdpi.com/journal/molecules

Supplementary material

Methylisoindigo and its bromo-derivatives are selective tyrosine kinase inhibitors, repressing cellular Stat3 activity, and target CD133+ cancer stem cells in PDAC

Jana Tegethoff[1+], Roland Bischoff[2+], Sawsan Saleh[1], Biljana Blagojevic[1], Karl-Heinz Merz[2], and Xinlai Cheng[1*]

[1] Department of Pharmacy and Molecular Biotechnology, Division of Pharmaceutical Biology, University of Heidelberg, Im Neuenheimer Feld 364, D-69120 Heidelberg, Germany.

[2] Department of Chemistry, Division of Food Chemistry and Toxicology, University of Kaiserslautern, Erwin-Schrödinger-Strasse 52, D-67663 Kaiserslautern, Germany.

+ Equal contribution

* Corresponding author: x.cheng@uni-heidelberg.de; Phone: +0049-6221-546431. Fax: +0049-6221-544884.

Received: / Accepted: / Published:

Table 1. Residual activity of human kinases at 20 µM meisoindigo performed by ProQinase (Freiburg, Germany).

#	*Kinase Name*	*Kinase Family*	*Residual activity at 20 µM*
**1**	ABL1	TK	88
**2**	ABL2	TK	66
**3**	ACK1	TK	69
**4**	ACV-R1	TKL	100
**5**	ACV-R1B	TKL	85
**6**	ACV-R2A	TKL	102
**7**	ACV-R2B	TKL	71
**8**	ACV-RL1	TKL	100
**9**	AKT1	AGC	104
**10**	AKT2	AGC	95
**11**	AKT3	AGC	106

**12**	ALK (GST-HIS-tag)	TK	53
**13**	AMPK-alpha1 aa1-550	CAMK	97
**14**	ARK5	CAMK	103
**15**	ASK1	STE	110
**16**	Aurora-A	OTHER	94
**17**	Aurora-B	OTHER	90
**18**	Aurora-C	OTHER	89
**19**	AXL	TK	88
**20**	BLK	TK	69
**21**	BMPR1A	TKL	83
**22**	BMX	TK	60
**23**	B-RAF	TKL	102
**24**	BRK	TK	39
**25**	BRSK1	CAMK	98
**26**	BTK	TK	82
**27**	CAMK1D	CAMK	98
**28**	CAMK2A	CAMK	78
**29**	CAMK2B	CAMK	91
**30**	CAMK2D	CAMK	84
**31**	CAMK4	CAMK	112
**32**	CAMKK1	OTHER	89
**33**	CAMKK2	OTHER	86
**34**	CDC42BPA	AGC	96
**35**	CDC42BPB	AGC	95
**36**	CDK1/CycA2	CMGC	96
**37**	CDK1/CycB1	CMGC	94
**38**	CDK1/CycE1	CMGC	99
**39**	CDK2/CycA2	CMGC	93
**40**	CDK2/CycE1	CMGC	90
**41**	CDK3/CycE1	CMGC	91
**42**	CDK4/CycD1	CMGC	100
**43**	CDK4/CycD3	CMGC	106
**44**	CDK5/p25NCK	CMGC	89
**45**	CDK5/p35NCK	CMGC	97
**46**	CDK6/CycD1	CMGC	86
**47**	CDK7/CycH/MAT1	CMGC	107
**48**	CDK8/CycC	CMGC	96
**49**	CDK9/CycK	CMGC	93
**50**	CDK9/CycT1	CMGC	109
**51**	CHK1	CAMK	109
**52**	CHK2	CAMK	91
**53**	CK1-alpha1	CK1	94

54	CK1-delta	CK1	92
55	CK1-epsilon	CK1	78
56	CK1-gamma1	CK1	91
57	CK1-gamma2	CK1	89
58	CK1-gamma3	CK1	95
59	CK2-alpha1	OTHER	103
60	CK2-alpha2	OTHER	99
61	CLK1	CMGC	82
62	CLK2	CMGC	112
63	CLK3	CMGC	63
64	CLK4	CMGC	84
65	COT	STE	100
66	CSF1-R	TK	57
67	CSK	TK	60
68	DAPK1	CAMK	89
69	DAPK2	CAMK	91
70	DAPK3	CAMK	84
71	DCAMKL2	CAMK	90
72	DDR2	TK	88
73	DMPK	AGC	99
74	DNA-PK	ATYP	111
75	DYRK1A	CMGC	97
76	DYRK1B	CMGC	91
77	DYRK2	CMGC	120
78	DYRK3	CMGC	97
79	DYRK4	CMGC	102
80	EEF2K	ATYPICAL	96
81	EGF-R	TK	61
82	EIF2AK2	OTHER	99
83	EIF2AK3	OTHER	106
84	EPHA1	TK	70
85	EPHA2	TK	42
86	EPHA3	TK	74
87	EPHA4	TK	99
88	EPHA5	TK	66
89	EPHA7	TK	60
90	EPHA8	TK	76
91	EPHB1	TK	40
92	EPHB2	TK	102
93	EPHB3	TK	35
94	EPHB4	TK	37
95	ERBB2	TK	89

96	ERBB4	TK	52
97	ERK1	CMGC	88
98	ERK2	CMGC	87
99	ERK7	CMGC	102
100	FAK aa2-1052	TK	76
101	FER	TK	24
102	FES	TK	35
103	FGF-R1	TK	62
104	FGF-R2	TK	28
105	FGF-R3	TK	40
106	FGF-R4	TK	52
107	FGR	TK	69
108	FLT3	TK	104
109	FRK	TK	67
110	FYN	TK	27
111	GRK2	AGC	98
112	GRK3	AGC	93
113	GRK4	AGC	73
114	GRK5	AGC	82
115	GRK6	AGC	90
116	GRK7	AGC	91
117	GSG2	OTHER	66
118	GSK3-alpha	CMGC	127
119	GSK3-beta	CMGC	94
120	HCK	TK	56
121	HIPK1	CMGC	99
122	HIPK2	CMGC	117
123	HIPK3	CMGC	89
124	HIPK4	CMGC	78
125	HRI	OTHER	107
126	IGF1-R	TK	20
127	IKK-alpha	OTHER	109
128	IKK-beta	OTHER	93
129	IKK-epsilon	OTHER	93
130	INS-R	TK	91
131	INSR-R	TK	99
132	IRAK1	TKL	73
133	IRAK4 (untagged)	TKL	91
134	ITK	TK	66
135	JAK1	TK	104
136	JAK2	TK	100
137	JAK3	TK	85

138	JNK1	CMGC	102
139	JNK2	CMGC	108
140	JNK3	CMGC	102
141	KIT	TK	88
142	LCK	TK	38
143	LIMK1	TKL	104
144	LIMK2	TKL	98
145	LRRK2	TKL	112
146	LTK	TK	57
147	LYN	TK	25
148	MAP3K1	STE	91
149	MAP3K10	STE	85
150	MAP3K11	STE	92
151	MAP3K7/MAP3K7IP1	STE	82
152	MAP3K9	STE	84
153	MAP4K2	STE	94
154	MAP4K4	STE	78
155	MAP4K5	STE	109
156	MAPKAPK2	CAMK	83
157	MAPKAPK3	CAMK	85
158	MAPKAPK5	CAMK	73
159	MARK1	CAMK	96
160	MARK2	CAMK	90
161	MARK3	CAMK	94
162	MARK4	CAMK	92
163	MATK	TK	69
164	MEK1	STE	85
165	MEK2	STE	94
166	MEKK2	STE	84
167	MEKK3	STE	76
168	MELK	CAMK	94
169	MERTK	TK	47
170	MET	TK	95
171	MINK1	STE	90
172	MKK6 S207D/T211D	STE	90
173	MKNK1	CAMK	98
174	MKNK2	CAMK	84
175	MST1	STE	80
176	MST2	STE	86
177	MST3	STE	90
178	MST4	STE	105
179	mTOR	ATYPICAL	92

180	MUSK	TK	91
181	MYLK	CAMK	83
182	MYLK2	CAMK	103
183	MYLK3	CAMK	125
184	NEK1	OTHER	94
185	NEK11	OTHER	94
186	NEK2	OTHER	110
187	NEK3	OTHER	89
188	NEK4	OTHER	85
189	NEK6	OTHER	110
190	NEK7	OTHER	79
191	NEK9	OTHER	88
192	NIK	STE	100
193	NLK	CMGC	107
194	p38-alpha	CMGC	81
195	p38-beta	CMGC	116
196	p38-delta	CMGC	79
197	p38-gamma	CMGC	106
198	PAK1	STE	107
199	PAK2	STE	105
200	PAK3	STE	105
201	PAK4	STE	139
202	PAK6	STE	85
203	PAK7	STE	85
204	PASK	CAMK	97
205	PBK	OTHER	94
206	PCTAIRE1/CycY	CMGC	120
207	PDGFR-alpha	TK	94
208	PDGFR-beta	TK	96
209	PDK1	AGC	68
210	PHKG1	CAMK	101
211	PHKG2	CAMK	133
212	PIM1	CAMK	91
213	PIM2	CAMK	90
214	PIM3	CAMK	92
215	PKA	AGC	88
216	PKC-alpha	AGC	119
217	PKC-beta1	AGC	106
218	PKC-beta2	AGC	111
219	PKC-delta	AGC	117
220	PKC-epsilon	AGC	84
221	PKC-eta	AGC	110

222	PKC-gamma	AGC	140
223	PKC-iota	AGC	103
224	PKC-mu	AGC	113
225	PKC-nu	AGC	97
226	PKC-theta	AGC	119
227	PKC-zeta	AGC	101
228	PLK1	OTHER	110
229	PLK3	OTHER	94
230	PRK1	AGC	90
231	PRK2	AGC	88
232	PRKD2	CAMK	95
233	PRKG1	AGC	81
234	PRKG2	AGC	88
235	PRKX	AGC	81
236	PYK2	TK	82
237	RAF1 Y340D/Y341D (untagged)	TKL	111
238	RET	TK	44
239	RIPK2	TKL	101
240	RIPK5	TKL	108
241	ROCK1	AGC	106
242	ROCK2	AGC	102
243	RON	TK	34
244	ROS	TK	89
245	RPS6KA1	AGC	102
246	RPS6KA2	AGC	90
247	RPS6KA3	AGC	94
248	RPS6KA4	AGC	106
249	RPS6KA5	AGC	84
250	RPS6KA6	AGC	111
251	S6K	AGC	81
252	S6K-beta	AGC	105
253	SAK	OTHER	93
254	SGK1	AGC	82
255	SGK2	AGC	99
256	SGK3	AGC	100
257	SLK	STE	110
258	SNARK	CAMK	67
259	SNF1LK2	CAMK	104
260	SNK	OTHER	102
261	SRC (GST-HIS-tag)	TK	38
262	SRMS	TK	70

263	SRPK1	CMGC	120
264	SRPK2	CMGC	105
265	STK17A	CAMK	79
266	STK23	CAMK	98
267	STK25	STE	95
268	STK33	CAMK	98
269	STK39	STE	138
270	SYK aa1-635	TK	77
271	TAOK2	STE	100
272	TAOK3	STE	109
273	TBK1	OTHER	91
274	TEC	TK	88
275	TGFB-R1	TKL	112
276	TGFB-R2	TKL	101
277	TIE2	TK	34
278	TLK1	AGC	111
279	TLK2	AGC	88
280	TRK-A	TK	69
281	TRK-B	TK	30
282	TRK-C	TK	64
283	TSF1	OTHER	89
284	TSK2	CAMK	96
285	TSSK1	CAMK	78
286	TTK	OTHER	87
287	TXK	TK	85
288	TYK2	TK	101
289	TYRO3	TK	59
290	VEGF-R1	TK	61
291	VEGF-R2	TK	37
292	VEGF-R3	TK	75
293	VRK1	CK1	103
294	WEE1	OTHER	110
295	WNK1	OTHER	104
296	WNK2	OTHER	92
297	WNK3	OTHER	91
298	YES	TK	36
299	ZAK	TKL	73
300	ZAP70	TK	83
